# Presumed granulomatosis with polyangiitis presenting with anterior scleritis and inflammatory ciliary body granuloma

**DOI:** 10.1186/s12348-025-00475-9

**Published:** 2025-03-13

**Authors:** Negin Yavari, Hashem Ghoraba, S. Saeed Mohammadi, Dalia El Feky, Irmak Karaca, Quan Dong Nguyen, Christopher Or

**Affiliations:** Spencer Center for Vision Research, Byers Eye Institute, Stanford University, Palo Alto, CA USA

**Keywords:** Anterior scleritis, Limited granulomatosis with polyangiitis, Ciliary body mass, Peripheral retinal mass, Rituximab, intravitreal dexamethasone implant

## Abstract

**Purpose:**

To present a case of presumed limited granulomatosis with polyangiitis (GPA) associated with anterior scleritis and ciliary body inflammatory granuloma which was treated with systemic rituximab (RTX), oral mycophenolate mofetil, and intravitreal (IVT) dexamethasone implant.

**Observations:**

We report a patient presenting with sectoral scleritis and ciliary body granuloma in the left eye. The patient also had a nasal sinus granuloma which was biopsied three times with negative results for malignancy and fungal infections. The patient underwent a diagnostic vitrectomy, which was also negative for lymphoma, bacterial and fungal infections. Subsequently, intravenous methylprednisolone and oral methotrexate were started, but significant improvement was achieved only following initiation of intravenous RTX, oral mycophenolate mofetil, and IVT dexamethasone implant.

**Conclusion:**

Therapeutic management of scleritis associated with limited GPA can be very challenging; early diagnosis can help to eliminate potential complications. Our result showed that RTX, mycophenolate mofetil, and IVT dexamethasone implant can be beneficial in treatment-resistant cases.

## Introduction

Granulomatosis with polyangiitis (GPA) is a rare systemic disease, with an annual incidence of 11.3 cases per million individuals [1]. Traditionally, the diagnosis of GPA relies on the presence of upper respiratory tract, lung, and kidney involvement, along with positive anti-neutrophil cytoplasmic antibodies (ANCA) or biopsy results. However, a limited form of GPA can present subtle characteristics including an absence of organ involvement and negative systemic diagnostic markers [2]. Given its rarity, diagnosing limited GPA poses significant challenges [2, 3]. To avoid misdiagnosis and treatment delay, clinicians must maintain vigilance and a high level of suspicion for limited GPA, as early intervention is crucial in preventing disease progression and associated complications [3–5].

Ophthalmologic involvement occurs in approximately 50% of GPA patients and represents a significant source of morbidity [6]. Various forms of ocular involvement have been documented, with scleral involvement being the most common [4, 7]. Scleral involvement associated with an inflammatory uveal granuloma is exceedingly rare, with only three documented cases in the literature, each showing variable treatment responses, and such rarity makes the condition a significant diagnostic and therapeutic challenge [8–10]. In this report, we present a rare case of presumed limited GPA with sectoral scleritis and ciliary body granuloma that exhibited resistance to treatment but was eventually controlled using rituximab (RTX), mycophenolate mofetil, and intravitreal (IVT) dexamethasone (Ozurdex^®^, Allergan, Inc., CA, USA) implant.

## Case presentation

A 55-year-old Asian woman was referred to the Uveitis Clinic at a tertiary eye center for the management of scleritis and iridocyclitis in her left eye, coinciding with the presence of a nasal sinus tumor. A few months before the referral, she experienced a vague pain in her left nostril, prompting imaging tests that revealed a nasal sinus mass. Two consecutive biopsies were conducted, both of which showed no signs of malignancy along with unremarkable laboratory evaluation. Two weeks after her initial diagnosis, the patient developed sectoral redness in her left eye, later diagnosed as anterior scleritis. The patient was referred to our institution for multidisciplinary management of her sinus mass and ocular inflammation.

**Fig. 1:**
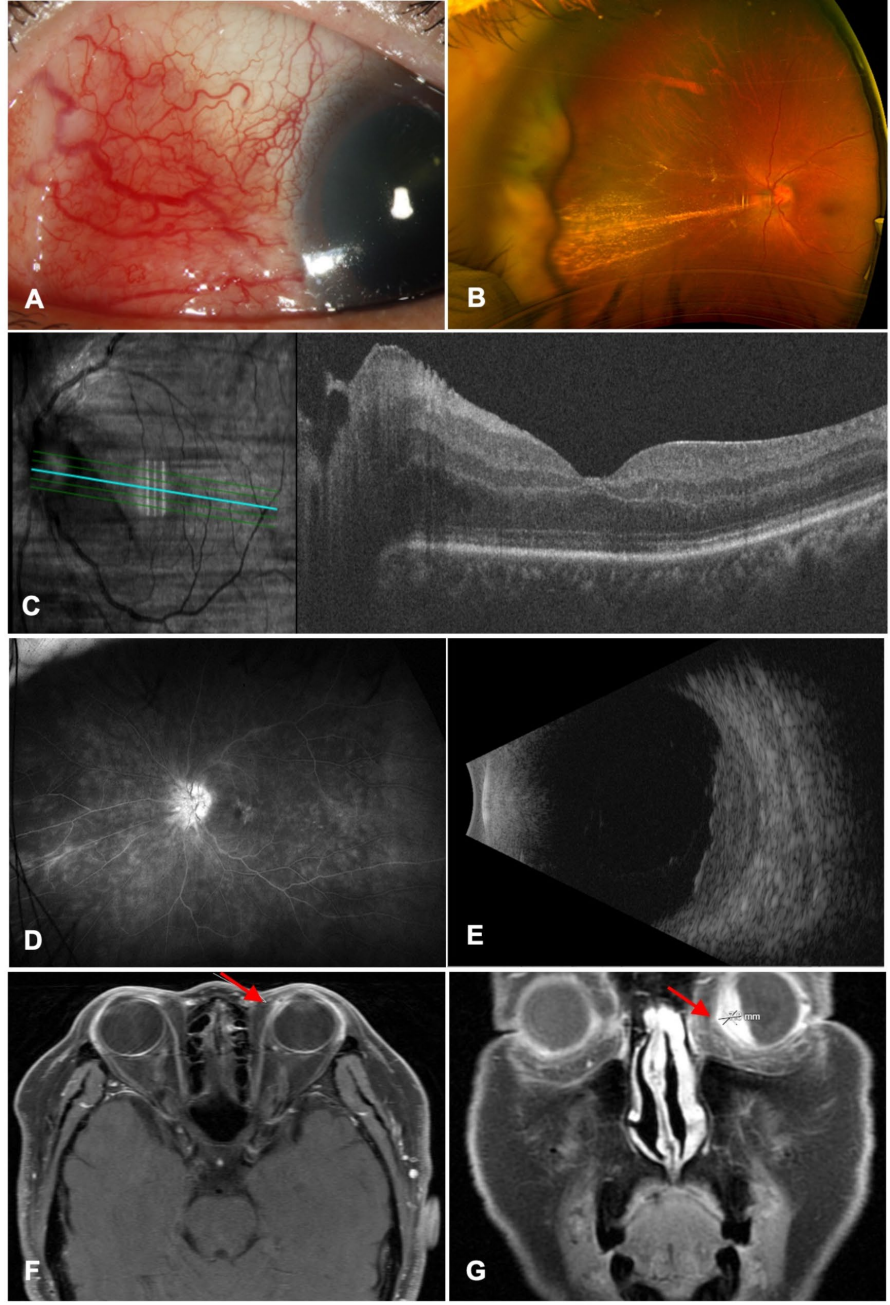
Selected images demonstrating finding of scleritis and ciliary body mass at presentation (left eye). **A)** Slit-lamp photograph showing 3 + scleral injection consistent with active anterior scleritis in nasal quadrant; **B)**  *Fundus photograph showing perivascular exudates and choroidal mass in the nasal retina*; **C)** Optical coherence topography showing macular thickening nasal to the fovea; **D)** Fluorescein angiography showing diffuse leakage of the optic disc and peripheral retinal vessels; **E)** Ultrasonography showing choroidal thickening with mild serous choroidal detachment; **F)** Magnetic resonance imaging showing enhancing lesion (arrow) in the left anterior nasal cavity with extra-nasal extension; **G)** Magnetic resonance imaging showing enhancing lesion along the medial ciliary body (arrow) in the left eye with extension to vitreous cavity

At initial evaluation, her best-corrected visual acuity (BCVA) was measured as 20/25 in the right eye and 20/60 in the left eye, and intraocular pressure was 18 in both eyes. Anterior segment examination revealed nasal sectoral scleritis (Fig. 1A), significant inflammation with 2 + cells and 2 + flare in the anterior chamber, as well as 3 + cells in the anterior vitreous in the left eye. Fundoscopic examination revealed 3 + vitreous haze, disc edema, and peripheral snow-banking (Fig. 1B). There was noticeable macular thickening on optical coherence tomography (OCT), without obvious subretinal or intraretinal fluids (Fig. 1C). Fluorescein angiography (FA) demonstrated disc leakage and peripheral retinal vasculitis (Fig. 1D). In addition, *ultrasonography revealed significant thickened choroidal mass with mild serous retinal detachment* (Fig. 1E). Magnetic resonance imaging (MRI) with contrast indicated an enhancing lesion in the left anterior nasal cavity with extra-nasal extension (Fig. 1F). It also revealed an enhancing lesion along the medial ciliary body in the left eye with extension to the vitreous cavity, which was suspicious for a neoplastic or inflammatory lesion (Fig. 1G). A third biopsy was performed on the nasal mass, revealing prominent mixed inflammation in the submucosa with no specific etiology, and mild reactive changes in the mucosa without cytologic atypia. The biopsy was negative for malignancy, bacterial and fungal infections. Laboratory evaluations also showed negative ANCA, antinuclear antibody (ANA), and rheumatoid factor (RF). Topical difluprednate was prescribed four times daily. Given the persistent severe inflammation and suspicion of lymphoma, a diagnostic vitrectomy was performed, which was negative for infection or malignancy. As a result, a diagnosis of presumed limited GPA was determined due to the manifestation of scleritis, presence of a nasal sinus granuloma, and absence of ANCA, ANA, and RF.

**Fig. 2:**
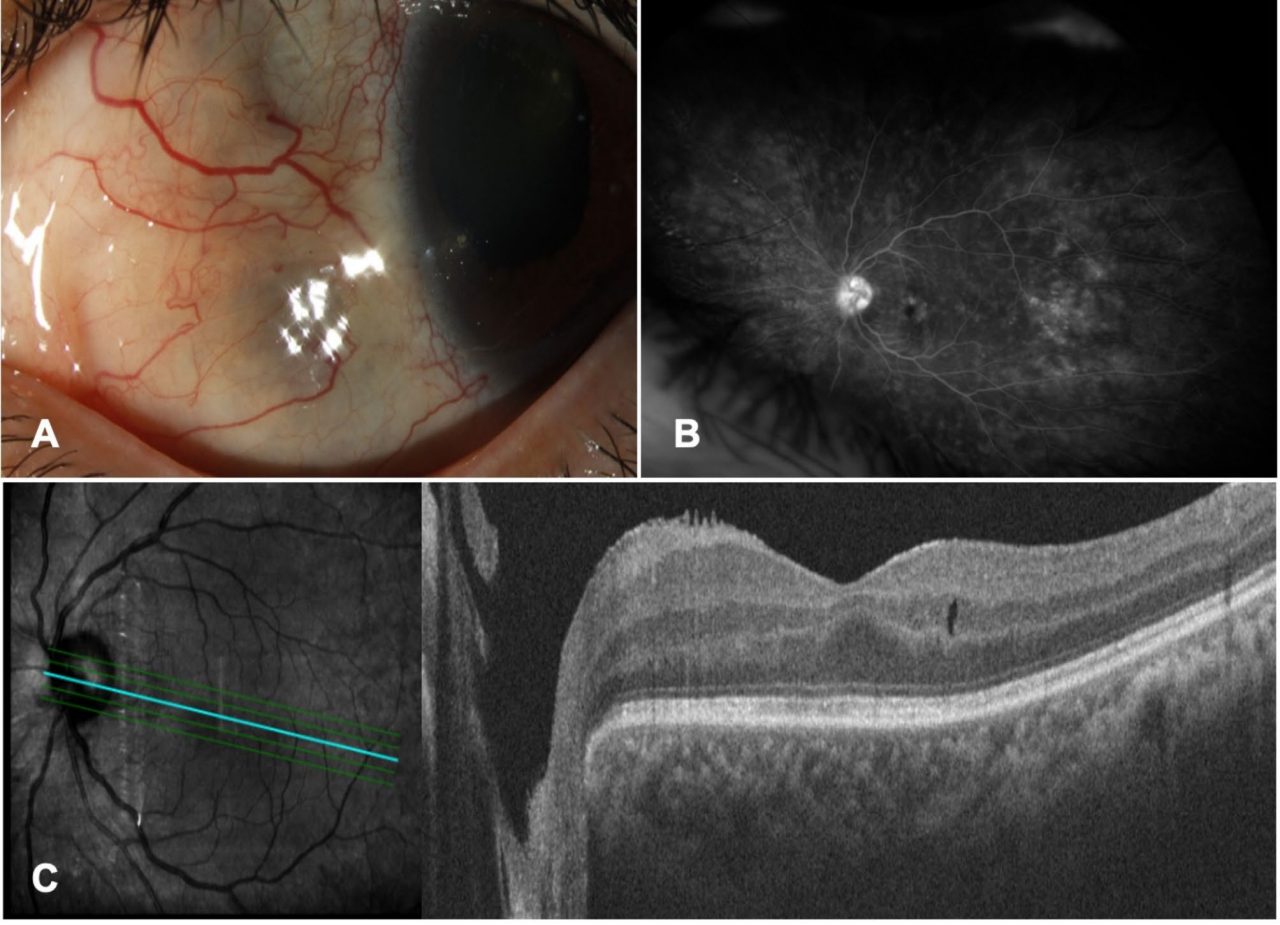
Selected images demonstrating scleritis and ciliary body mass before rituximab treatment (left eye). **A)** Slit-lamp photograph showing 1 + scleral injection with scleral thinning consistent with persistent anterior scleritis in nasal quadrant; **B)** Fluorescein angiography showing persistent leakage of peripheral retinal vessels with decreased optic disc leakage; **C)** Optical coherence topography showing decreased macular thickening nasal to the fovea

**Fig. 3:**
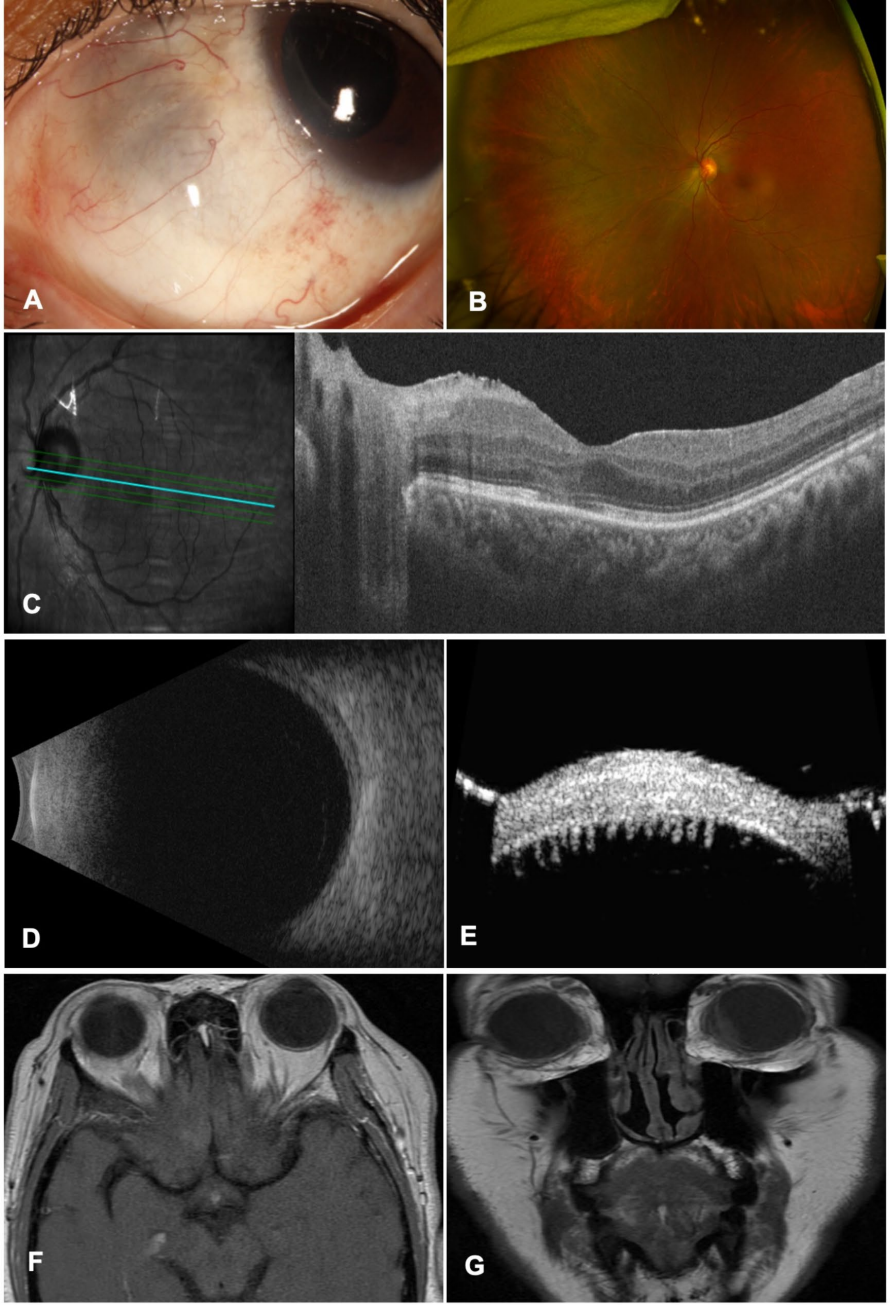
Selected images demonstrating improved scleritis and ciliary body mass after rituximab treatment (left eye). **A)** Slit-lamp photograph showing mild scleral thinning and resolved anterior scleritis in the nasal quadrant; **B)***Fundus photograph showing resolved perivascular exudates and choroidal mass in the nasal retina*; **C)** Optical coherence topography showing no intra- or subretinal fluid; **D)***Ultrabiomicroscopy (UBM) revealed* ciliary body and ciliary process atrophy at 9 PM; **E)***Ultrasonography revealed markedly decreased choroidal thickening with minimal residual subretinal fluid*; **F)** Magnetic resonance imaging showing resolution of lesion in the left anterior nasal cavity; **G)** Magnetic resonance imaging showing improvement in ciliary body mass

Patient was initiated on methotrexate (MTX) therapy 15 mg weekly, and three-monthly cycles of intravenous methylprednisolone 1000 mg followed by oral prednisone. Since persistent scleritis was noticed during follow-up visits, MTX dosage was increased to 25 mg weekly, and RTX infusion 1000 mg monthly was initiated. Imaging still revealed persistent scleritis with mild scleral thinning (Fig. 2A) and vasculitis and optic disc leakage (Fig. 2B), however OCT showed improvement of macular edema (Fig. 2C). As a result, MTX was switched to mycophenolate mofetil 1500 mg twice daily, which led to improvement of the scleritis and control of the condition (Fig. 3A). Given the stability of the ocular disease and presence of visually significant cataract, the patient underwent cataract surgery after a few months. During the post-operative follow up visit, rebound inflammation along with macular edema in the left eye was detected despite being on mycophenolate mofetil, prednisone, and RTX. Therefore, IVT dexamethasone implant was injected, which was successful in controlling the inflammation. In the last follow up, color fundus photo and OCT demonstrated stable inflammation (Fig. 3B, C) on RTX infusion every four months, and mycophenolate mofetil with visual acuity improved to 20/25 in the left eye. Repeated ultrasonography showing markedly decreased choroidal thickening with minimal residual subchoroidal fluid (Fig. 3D). Ultrasound biomicroscopy (UBM) exhibited ciliary body and ciliary process atrophy at 9 PM (Fig. 3E). MRI also showed resolution of lesion in the left nasal cavity (Fig. 3F) and improvement in medial ciliary body with minimal residual subchoroidal fluid in vitreous (Fig. 3G).

## Discussion

GPA, formerly Wegener’s granulomatosis, is a systemic vasculitis affecting small to medium-sized blood vessels, characterized by necrotizing granulomas. It commonly involves the upper and lower respiratory tracts and kidneys, potentially causing multisystem failure and death if untreated [2, 11, 12]. GPA is often associated with the presence of ANCA, which targets proteinase 3 (PR3) and myeloperoxidase (MPO). PR3-ANCA-associated GPA is frequently linked to scleritis [11, 13, 14]. Ocular manifestations, including scleritis and vasculitis, present as the first sign in 38.7% of cases. Patients with GPA could be classified into two categories: those with generalized disease (renal involvement) and those with limited disease (respiratory tract involvement) [15, 16].

The reported patient exhibited anterior scleritis, ciliary body granuloma, and a nasal mass. However, despite undergoing nasal biopsies and diagnostic vitrectomy, a definitive pathological diagnosis could not be determined. Therefore, considering the clinical presentation, along with the negative ANCA testing and absence of other pathognomonic findings, the patient was ultimately diagnosed with presumed limited ANCA-negative GPA, consistent with 2022 EULAR/ACR criteria [2, 3, 17]. The initial sign of GPA in the reported patient was a localized and limited sinus mass. Limited forms of GPA are particularly difficult to distinguish from neoplastic lesions [1, 3] as both may present as infiltrating masses in the maxillary sinus and nasopharyngeal space [3]. However, histological examination can often provide decisive diagnostic information to distinguish between these two conditions [3]. Recently, there has also been a report regarding limited GPA. These patients can present with negative serology and biopsy results, so called “double-negative”, making clinical manifestations crucial for diagnosis [18]. The main differential diagnoses considered in the clinical course included lymphoma, tuberculosis, sarcoidosis, immunoglobulin G4 related ophthalmic disease, and fungal infection, all of which were eliminated through multiple biopsies.

The treatment of ocular involvement in GPA is closely linked to the overall management of the underlying disease, involving systemic corticosteroids and cytotoxic medications [3, 13, 14]. Intravenous methylprednisolone can often result in significant improvement in limited GPA, although the benefits may not be long-lasting and there are systemic side effects with long-term use [1, 19]. Conventional therapy for GPA and scleritis has involved treating the vasculitis with a combination of high-dose corticosteroids and cyclophosphamide [1, 2, 13]. However, in cases of milder and limited forms of GPA and considering the high risk of adverse effects associated with cyclophosphamide, its use may not be practical [1, 13]. Currently, a combination therapy of glucocorticoids with either MTX or azathioprine has shown effectiveness in inducing and maintaining remission [1, 13]. Different studies have also demonstrated the efficacy of RTX in treating GPA with ocular involvement, particularly in refractory cases and in those with orbital granulomas [4, 7, 11, 14, 20–30].

Three cases of GPA associated with scleritis, and underlying choroidal granuloma have been published in the literature [8–10]. This rare presentation demonstrated treatment resistance. Out of the three cases documented in the literature, two were exceptionally severe and unresponsive to treatment, necessitating therapeutic enucleation due to complications such as necrotizing scleritis and subsequent loss of vision [8, 9]. Only one successful treatment approach, involving the administration of cyclophosphamide and prednisone, has been reported in the literature [10].

Few studies reported the use of IVT dexamethasone implant for refractory macular edema associated with GPA [31, 32]. Consistent with the literature, we were able to see improvement in inflammation and macular edema in our case after IVT dexamethasone injection [33, 34]. Even though the patient received multiple immunomodulatory treatments for her scleritis and vasculitis, the patient still had persistent inflammation, which was finally managed by dexamethasone implant. These findings indicate that IVT injection of dexamethasone implant can be safely and effectively used as a local therapy for non-necrotizing scleritis and vasculitis.

## Conclusion

In summary, the index report presents a case of presumed limited GPA involving sectoral scleritis and a ciliary body mass that were refractory to steroid and MTX treatment. Ultimately, the patient was successfully treated with intravenous RTX, oral mycophenolate mofetil, and IVT dexamethasone implant. Limited GPA, a rare disease with potentially severe consequences for vision, often exhibits ophthalmologic manifestations. Patients may initially present with scleritis and/or ocular/orbital granulomas.

Ophthalmologists need to maintain a heightened level of suspicion for GPA when ocular involvement(s) precede systemic symptoms. Additional imaging techniques like UBM or MRI of the orbit along with biopsies may be necessary to establish the diagnosis. Timely intervention is vital in the management of this disease, particularly those associated with scleritis and ciliary body granulomas. It is important to note that this condition can be refractory to various therapies, thus necessitating consideration of escalation of treatments if the desired clinical response cannot be achieved.

## Data Availability

No datasets were generated or analysed during the current study.

## Abbreviations

ANA: Antinuclear antibody
ANCA: Anti-neutrophil cytoplasmic antibodies
BCVA: Best corrected visual acuity
FA: Fluorescein angiography
GPA: Granulomatosis with polyangiitis
IVT: Intravitreal
MPO: Myeloperoxidase
MRI: Magnetic resonance imaging
MTX: Methotrexate
OCT: Optical coherence tomography
PRT3: Proteinase 3
RF: Rheumatoid factor
RTX: Rituximab
UBM: Ultrasound biomicroscopy
